# Light Scattering Calculations for Spherical Metallic Nanoparticles (Ag, Au) Coated by TCO (AZO, ITO, PEDOT:PSS) Shell

**DOI:** 10.3390/mi12091050

**Published:** 2021-08-30

**Authors:** Francesco Ruffino

**Affiliations:** Dipartimento di Fisica e Astronomia “Ettore Majorana”, Università di Catania, and CNR-IMM, Via S. Sofia 64, 95123 Catania, Italy; francesco.ruffino@ct.infn.it

**Keywords:** core–shell nanoparticles, transparent conductive oxide, light scattering, Mie theory, scattering efficiency, Au, Ag, AZO, ITO, PEDOT:PSS

## Abstract

Ag and Au nanostructures became increasingly interesting due to their localized surface plasmon resonance properties. These properties can be successfully exploited in order to enhance the light trapping in solar cell devices by appropriate light scattering phenomena. In solar cell applications, the Ag or Au nanoparticles are, usually, supported on or embedded in a thin transparent conductive oxide layer, mainly AZO and ITO for inorganic solar cells and PEDOT:PSS for organic solar cells. However, the light scattering properties strongly depend on the shape and size of the metal nanostructures and on the optical properties of the surrounding environment. Therefore, the systems need to be well designed to maximize scattering and minimize the light absorption within the metal nanoparticles. In this regard, this work reports, in particular, results concerning calculations, by using the Mie theory, of the angle-dependent light scattering intensity (I(θ)) for spherical Ag and Au nanoparticles coated by a shell of AZO or ITO or PEDOT:PSS. I(θ) and scattering efficiency Q_scatt_ for the spherical core–shell nanoparticles are calculated by changing the radius R of the spherical core (Ag or Au) and the thickness d of the shell (AZO, ITO, or PEDOT:PSS). For each combination of core–shell system, the evolution of I(θ) and Q_scatt_ with the core and shell sizes is drawn and comparisons between the various types of systems is drawn at parity of core and shell sizes. For simplicity, the analysis is limited to spherical core–shell nanoparticles so as to use the Mie theory and to perform analytically exact calculations. However, the results of the present work, even if simplified, can help in establishing the general effect of the core and shell sizes on the light scattering properties of the core–shell nanoparticles, essential to prepare the nanoparticles with desired structure appropriate to the application.

## 1. Introduction

Today, considerable attention is focused on plasmonic materials to improve the performance of optical-based devices for sensing and energy conversion applications [1,2,3]. In this sense, Ag and Au nanostructures have become interesting due to their localized surface plasmon resonance (LSPR) properties [1,2,3,4,5,6,7,8,9,10,11,12]. Light-trapping by Ag and Au nanostructures involves strong interaction of light with their conduction electrons. In this process, incident light excites the oscillation of the conduction of electrons at the surface of the metal particle of subwavelength size or at the interface between the metal particle and a supporting or embedding layer. When the natural frequency of the collectively oscillating electrons matches that of the incoming light, then an LSPR occurs. These plasmonic resonances can be used to preferentially scatter light into the active layer of a solar cell and, hence, to enhance the free carriers’ generation [13]. More generally, light trapping metal nanoparticles allow the long-wavelength radiation to travel in solar cells to distances much longer than the device thickness, therefore increasing the probability of light absorption. In any case, this involves the opportune integration of metal nanoparticles into the cell structure. Therefore, the exploitation of LSPR of Ag and Au nanostructures is one of the key approaches to increase the optical absorption, by light trapping, in thin film Si solar cells, organic solar cells, and new-generation solar cells [1,2,3,4,5,13,14]. So, several schemes for functional devices incorporating plasmonic Ag and Au nanoparticles were recently proposed [15,16,17,18,19,20,21,22,23,24,25,26,27,28,29,30,31,32,33,34]. Generally, this can be achieved by the scattering of light, by the metal nanoparticles, at the cell’s interfaces, either by transmission at the top interfaces or by reflection at the rear ones [34]. Ag and Au nanostructures are interesting for such applications since they can plasmonically scatter light due to their strong plasmon resonances in the visible to near-infrared spectral region depending on the control of their size and shape (they have a large scattering cross-section but a low absorption cross-section in the visible and near infrared region of the solar spectrum). However, the resonant optical properties of the metal nanoparticles depend strongly on the geometry and size of the nanoparticles, as well as on the surrounding environment [1,2,3,4,5]. The absorption and scattering cross-sections for spherical nanoparticles depend strongly on the radius of the particle and the dielectric constants of the particle and surrounding medium. Therefore, it is of paramount importance to select a medium with appropriate dielectric constant to produce the desired plasmonic resonances. For specific resulting performances, so, the system needs to be geometrically optimized to maximize scattering and minimize light absorption within the metal nanoparticles across the wavelength range of interest. In solar cell applications, the Ag or Au nanoparticles are usually supported on or embedded in a thin transparent conductive oxide (TCO) layer [15,16,17,18,19,20,21,22,23,24,25,26,27,28,29,30,31,32,33,34]. TCOs are widely used as transparent electrodes for a variety of solar cell devices [35]. For example, in heterojunction Si wafer solar cells, TCO layers are used for carrier transport and provide good antireflection component. For these applications, thus, it is a challenge to optimize both the electrical and the optical performances of TCOs [17]. Indium tin oxide (ITO) is the most-used TCO material for solar cells, due to its excellent electrical and optical properties [35]. However, due to the high cost of ITO, there is an increasing interest in more cost-effective materials such as aluminum-doped zinc oxide (AZO) [35]. In addition, in organic solar cell devices, poly-(3,4-ethylenedioxythiophene):poly(styrenesulfonate) (PEDOT:PSS) is the most-used TCO material [26], also in view of production of flexible devices. These materials have, typically, ε_m_ ≈ 4 and could serve as supporting or embedding medium for Ag and Au nanoparticles. For example, Ag particles with a diameter of 70 nm embedded in a TCO would be very suitable as light scattering elements in solar cells [17]. Their extinction efficiency is about 10, which means that as a first approximation a film of such particles with a surface coverage of only 10% would be able to absorb or scatter all incident light [17]. For wavelength λ > 500 nm they have a radiative efficiency of about 0.95, meaning that they scatter 95% and absorb only 5% of the light [17]. Particles of this size are particularly interesting for light trapping in hydrogenated amorphous silicon (a-Si:H) solar cells because they have a high extinction efficiency in the wavelength range where light trapping is required as a-Si:H is weakly absorbing in that wavelength range.

On the basis of these considerations, this work reports results concerning calculations, by using the Mie theory, of the angle-dependent light scattering intensity (I(θ)) for spherical Ag and Au nanoparticles coated by TCO shell as AZO, ITO, PEDOT:PSS due to their potential applications in plasmonic solar cell devices. In particular, I(θ) and scattering efficiency Q_scatt_ for the spherical core–shell nanoparticles are calculated by changing the radius R of the spherical core (Ag or Au) and the thickness d of the shell (AZO, ITO, or PEDOT:PSS). For each combination of core–shell system, the evolution of I(θ) and Q_scatt_ with the core and shell sizes is drawn and comparisons between the various types of systems is drawn fixing the core and shell sizes. For simplicity, the analysis is limited to spherical core–shell nanoparticles so we use the Mie theory for analytical exact calculations. From the experimental point of view, non-spherical metal nanostructures (complex-morphology nanostructures) present plasmonic hot-spots in correspondence of apex-shaped geometries or double-bands plasmonic absorption as in nanorods [1,2,3,4,9]. In these systems, the interaction of the electromagnetic radiation with the metallic nanostructures is more complicated and can results in additional effects. However, the results of the present work, even if simplified, could help in establishing the general effect of the core and shell sizes on the light scattering properties of the core–shell nanoparticles, essential to select specific size conditions for the nanoparticles so to obtain desired structures with peculiar properties appropriate to the application.

Even if a real theoretical innovation cannot be claimed in the approach for the calculations presented here, the scientific insight addressed by the present work is strongly connected to the potential technological applications, mainly related to novel solar cell devices. In this regard, the results of the calculations here presented could acquire value and utility for researchers comparing these calculation results to real situations and experimental data. Hence, the present calculation results can be applied, in combination to experimental results, to choose strategies for the optimization of the structure of the core–shell nanoparticles to reach a specific light scattering behavior for desired performances in optical-based devices. Within this framework, the present paper continues a series of papers on the use of the Scatlab software to compute the light scattering properties of metal-based core–shell nanoparticles [36,37] and investigates the corresponding possible technological applications.

## 2. Calculations and Results

A complete theory of the scattering and absorption of electromagnetic radiation by a homogeneous sphere was developed by Gustav Mie [38]. The Mie theory is an exact solution to the Maxwell Equations (1)–(4), in SI units, for the macroscopic electromagnetic field at interior points in matter and is valid for spherical particles of any size embedded in a homogeneous medium [39,40,41,42,43],

(1) $$\nabla \cdot D=\rho_{F}$$

(2) $$\nabla \times E+\frac{\partial B}{\partial t}=0$$

(3) ∇·B=0

(4) $$\nabla \times H=J_{F}+\frac{\partial D}{\partial t}$$

where **E** is the electric field, **B** the magnetic induction, ρ_F_ the charge density, **J**_F_ the current density, **D** the electric displacement, and **H** the magnetic field defined, respectively, as

(5) $$D=\varepsilon_{0}E+P$$

(6) $$H=\frac{B}{\mu_{0}}-M$$

where **P** is the electric polarization (average electric dipole moment per unit volume), **M** the magnetization (average magnetic dipole moment per unit volume), ε_0_ the permittivity, and μ_0_ the permeability of the free space. Equations (1)–(6) are not sufficient in themselves; they must be supplemented with constitutive relations which are assumed to have the form **J**_F_ = σ**E**, **B** = μ**H**, **P** = ε_0_χ**E** with σ the conductivity, μ the permeability, and χ the electric susceptibility. 

The Mie’s approach is based on the expansion of the internal and scattered fields into a set of normal modes described by vector harmonics [39,40,41,42,43]. The quasi-static results valid for subwavelength spheres are then recovered by a power series expansion of the absorption and scattering coefficients and taking into in account only the first term. Therefore, the Mie’s theory is applicable only to spherical particles (spherical symmetry). The Mie’s theory was generalized to analytically investigate the electromagnetic radiation scattering and absorption properties by multi-layered spherical particles [42,43] which is the case of the interaction of spherical core–shell nanoparticles with the electromagnetic radiation. 

The main parameter to characterize the scattering process of the electromagnetic radiation from a particle is the scattering cross section σ, defined as the ratio between the total radiation scattered power to the radiation intensity, σ = W/I_0_ being I_0_ (energy/(area)(time)) the intensity of a plane electromagnetic wave impinging on the spherical particle and W (energy/time) the total (i.e., integrated over all directions) power of the wave scattered by the particle. Usually, what is experimentally measured is the scattering efficiency defined as Q_scatt_ = σ/πR^2^ being the scattering cross section σ normalized to the geometrical section πR^2^ (area of a circle of radius R) of the spherical particle of radius R. Exploiting the definition of Q_scatt_, a dimensionless parameter, the electromagnetic radiation scattering properties of spherical particles with different sizes can be directly compared. Q_scatt_ can exceed 1 for a particle since in addition to scattering radiation incident on its geometrical cross section, the particle also diffracts radiation at its edges, so that it can behave as a larger particle than its geometrical cross section. In order to calculate σ for a homogeneous spherical particle, the Maxwell’s equations can be analytically solved considering a plane wave incident on the particle. Then, Q_scatt_ can be calculated. Similarly, the Maxwell’s equations can be solved considering a layered spherical particle on which a plan electromagnetic wave is incident: if the particle is formed by N layers (from 1, the core, to N, the outmost shell), with each layer having radius R_i_ (R_1_ the radius of the core, R_N_ the thickness of the outmost shell) and refractive index n_i_ (n_1_ the refractive index of the core, n_N_ the refractive index of the outmost shell, n_b_ the refractive index of a matrix embedding the layered particle, n the refractive index of the medium that the wave is traveling), then the incident, scattered, and internal fields can be expanded as a superposition of vector spherical harmonics (thanks to the spherical symmetry). 

This approach is exploited by algorithms used in various software to carry out the calculations [44] as, for example, Pyscatmech, Lorentz-Mie Scattering, Pyshs, Stratify, Miepython, Pymiescatt, Menp, and ScatLab. In the present work the Scatlab 1.2.111 software [45] is used to calculate the angle-dependent intensities (I(θ)) and the scattering efficiency (Q_scatt_) for the spherical core–shell particles Ag/AZO, Au/AZO, Ag/ITO, Au/ITO, Ag/PEDOT:PSS, and Au/PEDOT:PSS by changing the size of the core and of the shell and by fixing the wavelength of the incident electromagnetic wave to λ = 550 nm (the center of the visible spectrum, in view of visible light induced phenomena and visible light-based applications). ScatLab is a software developed to perform electromagnetic scattering simulations based on classical Mie theory solution. It is designed to meet windows type guidelines. The computation capabilities of Scatlab are based on the possibility to calculate scattered intensity polar diagrams for coated and uncoated spherical particles, scattered intensity versus radius graphs for homogeneous spherical particles, polarization rate versus radius graph for homogeneous spherical particles, extinction, scattering and backscattering cross section graphs, polarization rate versus damping rate graph, angle depolarization graphs, near field imaging for homogeneous spherical particles, near field average scattered intensity versus radius graphs for homogeneous spherical particles, Lorentz and Drude dielectric function implementation for refractive index calculation, and more other. As generally described above, the Scalab software is one type of calculator (based on the Mie theory), which consider an incident plane wave as represented by an infinite combination of spherical harmonics. Their amplitudes depend on the polarization and the direction of the incident wave and are given in general case by analytical formulae. The advantage of such representation is in that each such harmonics is scattered as a single spherical outgoing harmonics which amplitude depends on the particle radius and refractive indices and is prescribed by coefficient given by an analytical expression. Since each scattered harmonics propagates independently, the total scattered power is found as the sum of particular powers in all scattered harmonics. In addition, the solutions of the calculations are dependent on the specific boundary conditions under which the program operates. Generally, the following conditions are imposed: (a) interface conditions on the boundary between the spherical particle and the environment (which allow to relate the expansion coefficients of the incident, internal, and scattered fields); (b) the condition that the solution is bounded at the origin; (c) for a scattered field, the asymptotics at infinity corresponds to a diverging spherical wave. Values commonly calculated by software using Mie theory, as Scatlab, include efficiency coefficients for extinction, scattering, and absorption. The solutions solve for an infinite harmonic series, and provide as output the calculation of the scattering phase function, extinction, scattering, and absorption efficiencies. These efficiency coefficients are ratios of the cross-section of the respective process to the particle area. The dependence of the scattering cross-section on the wavelength and the contribution of specific resonances strongly depends on the particle material. For example, for a Au particle with a radius of 100 nm, the contribution of the electric dipole to scattering predominates in the optical range, while for a Si particle there are pronounced magnetic dipole and quadrupole resonances. For metal particles, the peak visible in the scattering cross-section is the localized plasmon resonance. In the limit of small particles or long wavelengths, the electric dipole contribution dominates in the scattering cross-section. Hence, the selection of the values for the real part and imaginary part of the refractive index of the material composing the particle for each analyzed wavelength is important to obtain reliable results. However, overall, the Scatlab software was widely used the calculate the optical properties of several typologies of spherical single-component or multilayered particles with excellent results since in agreement with the experimental results within the range for which the Mie theory holds and for which the experimental conditions adhere to the validity hypothesis for the theory [43,46,47,48,49]. 

In particular, we exploit the Scatlab capabilities to calculate the light scattering properties of the for the spherical bimetallic core–shell nanoparticles made by Ag/AZO, Au/AZO, Ag/ITO, Au/ITO, Ag/PEDOT:PSS, and Au/PEDOT:PSS for various combinations of the core radius and shell width: hence, within the capabilities and limits of the Scatlab software, the additional scientific inside of the present work relies in the application of a freely available software to functional nanomaterials with potential interesting applications and in the setting of a general framework connecting the nanoparticles geometry to their light scattering characteristics. Therefore, our work is in the line with the computational design of the best geometries for the core–shell nanoparticles for improving the performance of solar cells devices where Ag or Au particles, in combination with TCOs, are used to enhance the light scattering efficiency. 

In order to perform the calculations, the Scatlab software requires, as input parameters, values for the real part, n, and imaginary part, k, of the refractive index of the materials composing the particle and of the matrix where the particle is embedded (and corresponding to the chosen wavelength of the incident electromagnetic radiation), and values for the particle core radius R and particle shell width d. Regarding the metals here investigated, the values for n and k used for the calculations are reported in Table 1 (for λ = 550 nm) as extracted by ref. [50]. The core–shell particles are supposed to be placed in air so that n = 1 and k = 0 for the matrix embedding the particles.

**Figure 1:**
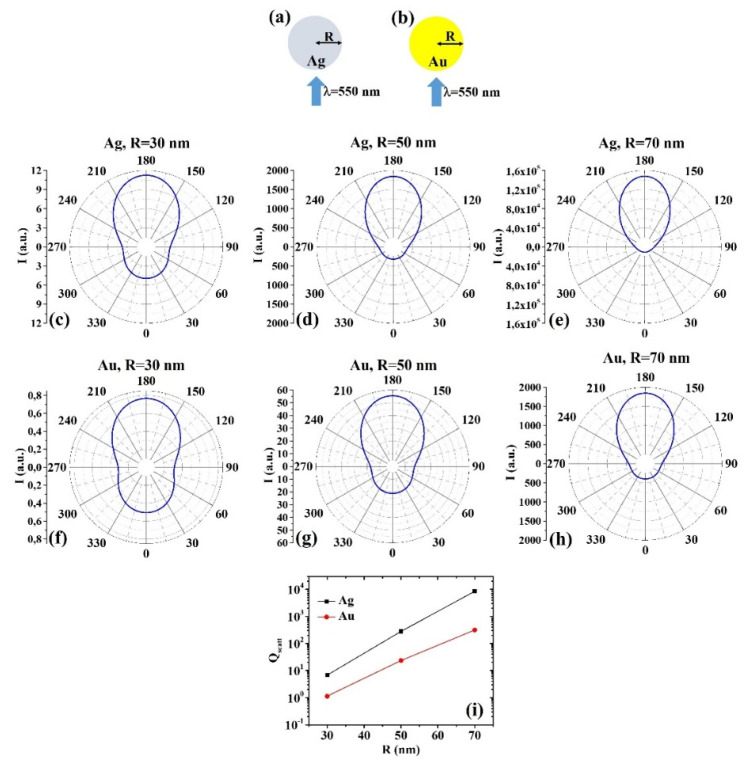
(**a**,**b**) Scheme of the structure of the simulated Ag (**a**) and Au (**b**) pure spherical particle with radius R and electromagnetic radiation of wavelength λ = 550 nm impinging on the particle from 0°. (**c**–**e**) Calculated polar diagrams for the intensity of the scattered light from the Ag spherical particle changing the radius R from 30 nm to 70 nm. (**f**–**h**) Calculated polar diagrams for the intensity of the scattered light from the Au spherical particle changing the radius R from 30 nm to 70 nm. (**i**) Calculated scattering efficiency for the light (wavelength λ = 550 nm) scattering process of the Ag (black dots) and Au (red dots) spherical particle increasing the particle radius from 30 nm to 70 nm.

**Figure 2:**
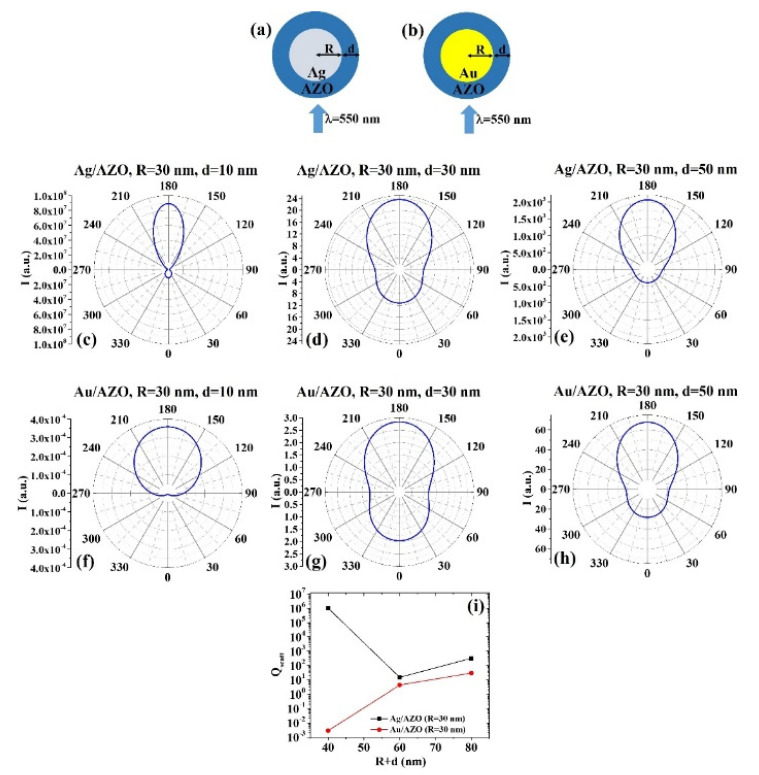
(**a**,**b**) Scheme of the structure of the simulated Ag/AZO (**a**) and Au/AZO (**b**) core–shell spherical particle with core radius R and shell width d and electromagnetic radiation of wavelength λ = 550 nm impinging on the particle from 0°. (**c**–**e**) Calculated polar diagrams for the intensity of the scattered light from the Ag/AZO core–shell spherical particle fixing the Ag core radius R to 30 nm and increasing the AZO shell width from 10 nm to 50 nm. (**f**–**h**) Calculated polar diagrams for the intensity of the scattered light from the Au/AZO core–shell spherical particle fixing the Au core radius R to 30 nm and increasing the AZO shell width from 10 nm to 50 nm. (**i**) Calculated scattering efficiency for the light (wavelength λ = 550 nm) scattering process of the Ag/AZO (black dots) and Au/AZO (red dots) core–shell spherical particles fixing the core radius to R = 30 nm and increasing the AZO shell width d from 10 nm to 50 nm.

**Figure 3:**
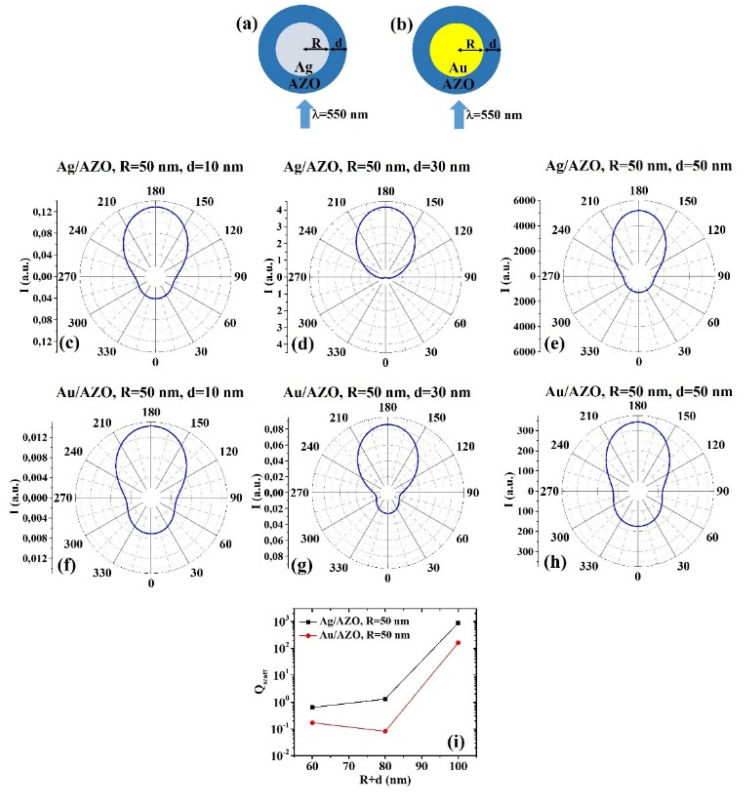
(**a**,**b**) Scheme of the structure of the simulated Ag/AZO (**a**) and Au/AZO (**b**) core–shell spherical particle with core radius R and shell width d and electromagnetic radiation of wavelength λ = 550 nm impinging on the particle from 0°. (**c**–**e**) Calculated polar diagrams for the intensity of the scattered light from the Ag/AZO core–shell spherical particle fixing the Ag core radius R to 50 nm and increasing the AZO shell width from 10 nm to 50 nm. (**f**–**h**) Calculated polar diagrams for the intensity of the scattered light from the Au/AZO core–shell spherical particle fixing the Au core radius R to 50 nm and increasing the AZO shell width from 10 nm to 50 nm. (**i**) Calculated scattering efficiency for the light (wavelength λ = 550 nm) scattering process of the Ag/AZO (black dots) and Au/AZO (red dots) core–shell spherical particles fixing the core radius to R = 50 nm and increasing the AZO shell width d from 10 nm to 50 nm.

**Figure 4:**
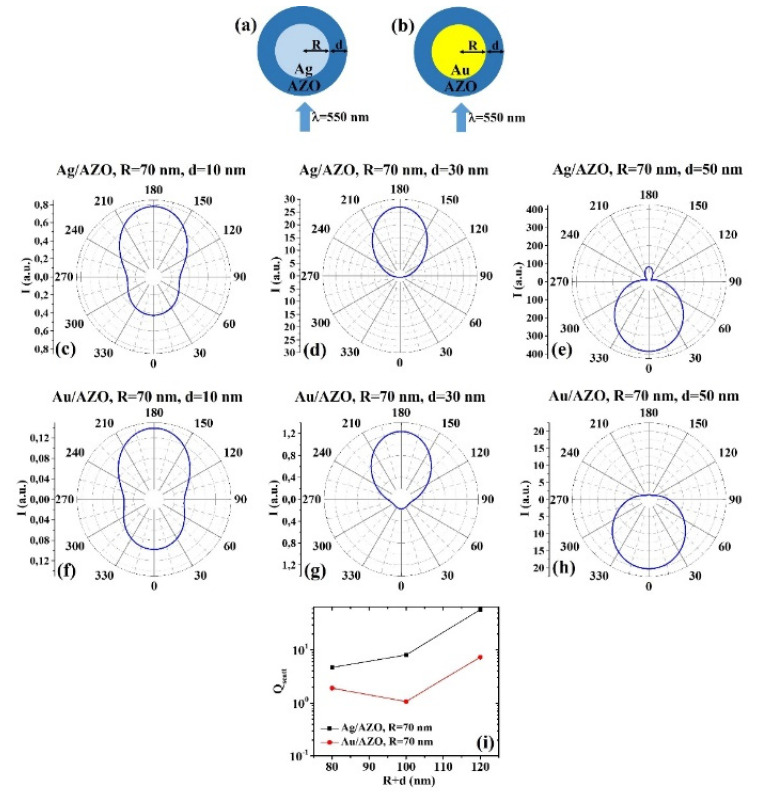
(**a**,**b**) Scheme of the structure of the simulated Ag/AZO (**a**) and Au/AZO (**b**) core–shell spherical particle with core radius R and shell width d and electromagnetic radiation of wavelength λ = 550 nm impinging on the particle from 0°. (**c**–**e**) Calculated polar diagrams for the intensity of the scattered light from the Ag/AZO core–shell spherical particle fixing the Ag core radius R to 70 nm and increasing the AZO shell width from 10 nm to 50 nm. (**f**–**h**) Calculated polar diagrams for the intensity of the scattered light from the Au/AZO core–shell spherical particle fixing the Au core radius R to 70 nm and increasing the AZO shell width from 10 nm to 50 nm. (**i**) Calculated scattering efficiency for the light (wavelength λ = 550 nm) scattering process of the Ag/AZO (black dots) and Au/AZO (red dots) core–shell spherical particles fixing the core radius to R = 70 nm and increasing the AZO shell width d from 10 nm to 50 nm.

**Figure 5:**
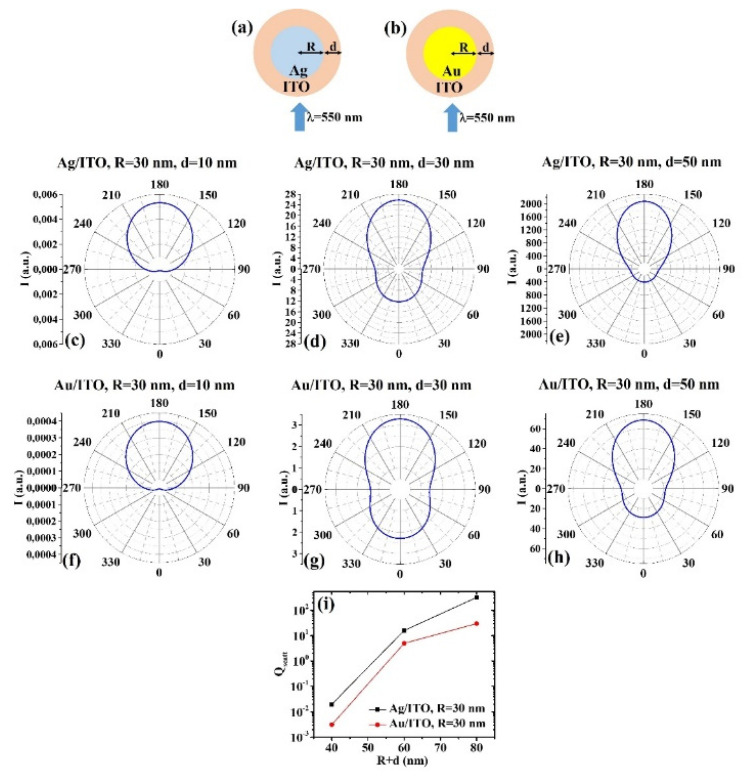
(**a**,**b**) Scheme of the structure of the simulated Ag/ITO (**a**) and Au/ITO (**b**) core–shell spherical particle with core radius R and shell width d and electromagnetic radiation of wavelength λ = 550 nm impinging on the particle from 0°. (**c**–**e**) Calculated polar diagrams for the intensity of the scattered light from the Ag/ITO core–shell spherical particle fixing the Ag core radius R to 30 nm and increasing the ITO shell width from 10 nm to 50 nm. (**f**–**h**) Calculated polar diagrams for the intensity of the scattered light from the Au/ITO core–shell spherical particle fixing the Au core radius R to 30 nm and increasing the ITO shell width from 10 nm to 50 nm. (**i**) Calculated scattering efficiency for the light (wavelength λ = 550 nm) scattering process of the Ag/ITO (black dots) and Au/ITO (red dots) core–shell spherical particles fixing the core radius to R = 30 nm and increasing the ITO shell width d from 10 nm to 50 nm.

**Figure 6:**
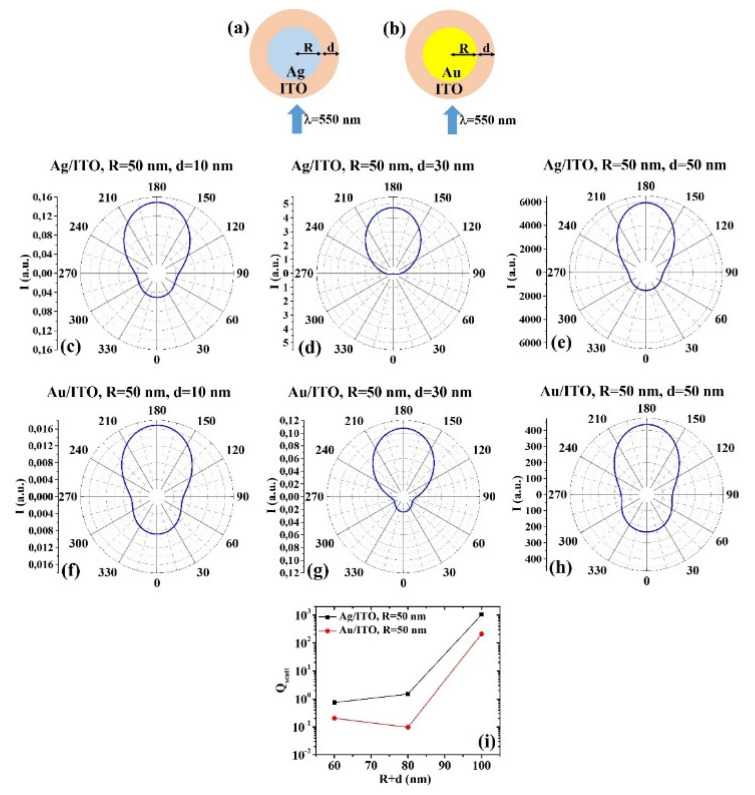
(**a**,**b**) Scheme of the structure of the simulated Ag/ITO (**a**) and Au/ITO (**b**) core–shell spherical particle with core radius R and shell width d and electromagnetic radiation of wavelength λ = 550 nm impinging on the particle from 0°. (**c**–**e**) Calculated polar diagrams for the intensity of the scattered light from the Ag/ITO core–shell spherical particle fixing the Ag core radius R to 50 nm and increasing the ITO shell width from 10 nm to 50 nm. (**f**–**h**) Calculated polar diagrams for the intensity of the scattered light from the Au/ITO core–shell spherical particle fixing the Au core radius R to 50 nm and increasing the ITO shell width from 10 nm to 50 nm. (**i**) Calculated scattering efficiency for the light (wavelength λ = 550 nm) scattering process of the Ag/ITO (black dots) and Au/ITO (red dots) core–shell spherical particles fixing the core radius to R = 50 nm and increasing the ITO shell width d from 10 nm to 50 nm.

**Figure 7:**
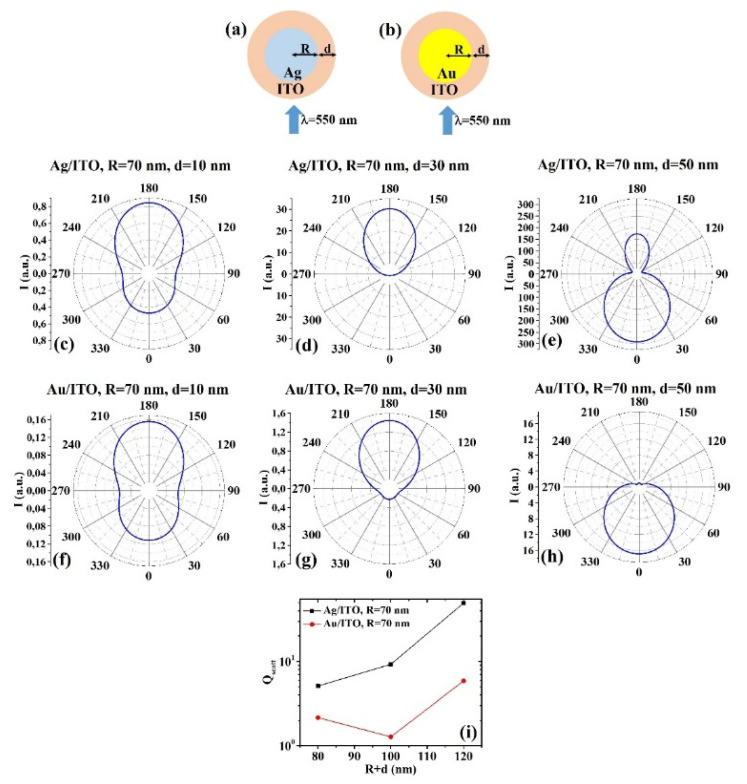
(**a**,**b**) Scheme of the structure of the simulated Ag/ITO (**a**) and Au/ITO (**b**) core–shell spherical particle with core radius R and shell width d and electromagnetic radiation of wavelength λ = 550 nm impinging on the particle from 0°. (**c**–**e**) Calculated polar diagrams for the intensity of the scattered light from the Ag/ITO core–shell spherical particle fixing the Ag core radius R to 70 nm and increasing the ITO shell width from 10 nm to 50 nm. (**f**–**h**) Calculated polar diagrams for the intensity of the scattered light from the Au/ITO core–shell spherical particle fixing the Au core radius R to 70 nm and increasing the ITO shell width from 10 nm to 50 nm. (**i**) Calculated scattering efficiency for the light (wavelength λ = 550 nm) scattering process of the Ag/ITO (black dots) and Au/ITO (red dots) core–shell spherical particles fixing the core radius to R = 70 nm and increasing the ITO shell width d from 10 nm to 50 nm.

**Figure 8:**
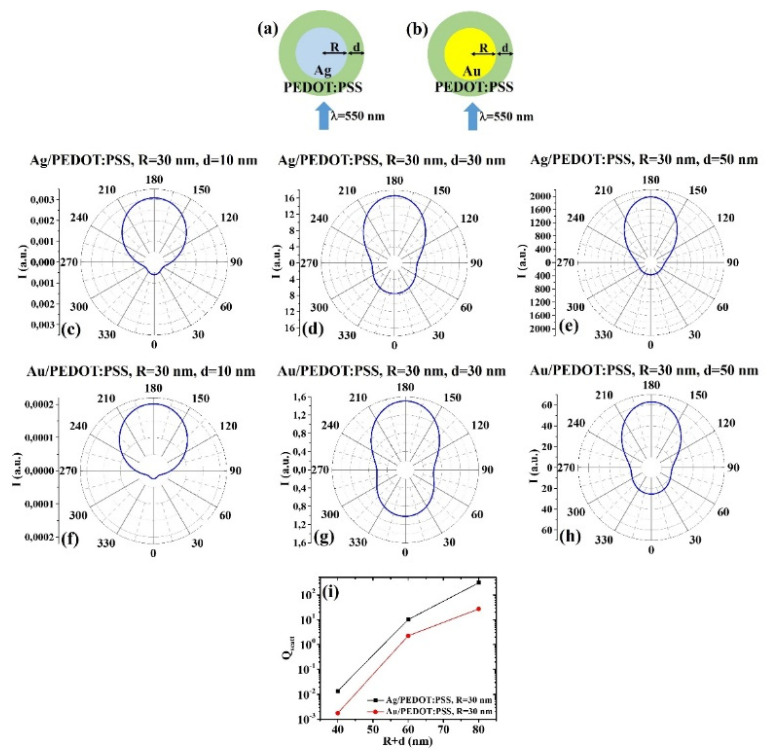
(**a**,**b**) Scheme of the structure of the simulated Ag/PEDOT:PSS (**a**) and Au/PEDOT:PSS (**b**) core–shell spherical particle with core radius R and shell width d and electromagnetic radiation of wavelength λ = 550 nm impinging on the particle from 0°. (**c**–**e**) Calculated polar diagrams for the intensity of the scattered light from the Ag/PEDOT:PSS core–shell spherical particle fixing the Ag core radius R to 30 nm and increasing the PEDOT:PSS shell width from 10 nm to 50 nm. (**f**–**h**) Calculated polar diagrams for the intensity of the scattered light from the Au/PEDOT:PSS core–shell spherical particle fixing the Au core radius R to 30 nm and increasing the PEDOT:PSS shell width from 10 nm to 50 nm. (**i**) Calculated scattering efficiency for the light (wavelength λ = 550 nm) scattering process of the Ag/PEDOT:PSS (black dots) and Au/PEDOT:PSS (red dots) core–shell spherical particles fixing the core radius to R = 30 nm and increasing the PEDOT:PSS shell width d from 10 nm to 50 nm.

**Figure 9:**
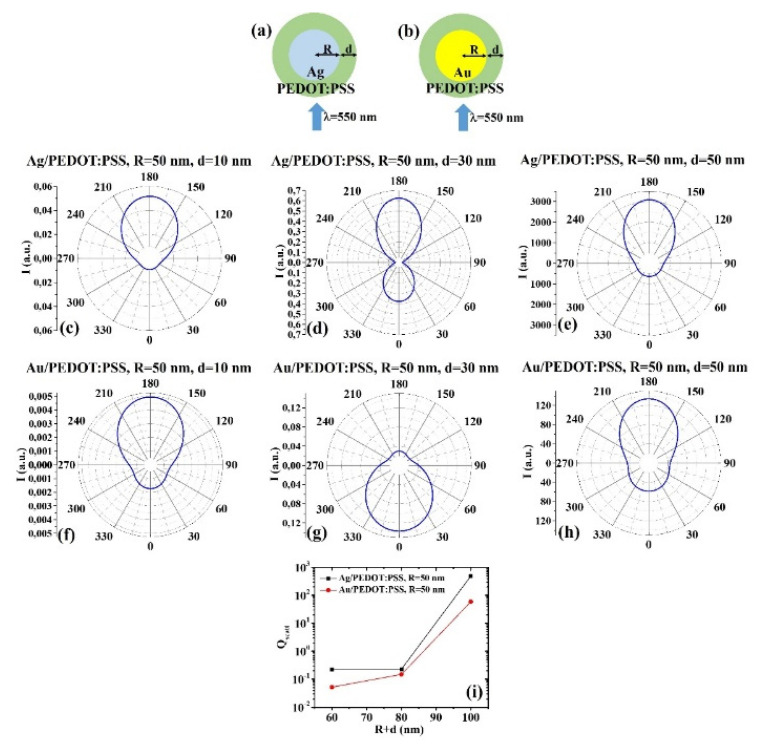
(**a**,**b**) Scheme of the structure of the simulated Ag/PEDOT:PSS (**a**) and Au/PEDOT:PSS (**b**) core–shell spherical particle with core radius R and shell width d and electromagnetic radiation of wavelength λ = 550 nm impinging on the particle from 0°. (**c**–**e**) Calculated polar diagrams for the intensity of the scattered light from the Ag/PEDOT:PSS core–shell spherical particle fixing the Ag core radius R to 50 nm and increasing the PEDOT:PSS shell width from 10 nm to 50 nm. (**f**–**h**) Calculated polar diagrams for the intensity of the scattered light from the Au/PEDOT:PSS core–shell spherical particle fixing the Au core radius R to 50 nm and increasing the PEDOT:PSS shell width from 10 nm to 50 nm. (**i**) Calculated scattering efficiency for the light (wavelength λ = 550 nm) scattering process of the Ag/PEDOT:PSS (black dots) and Au/PEDOT:PSS (red dots) core–shell spherical particles fixing the core radius to R = 50 nm and increasing the PEDOT:PSS shell width d from 10 nm to 50 nm.

**Figure 10:**
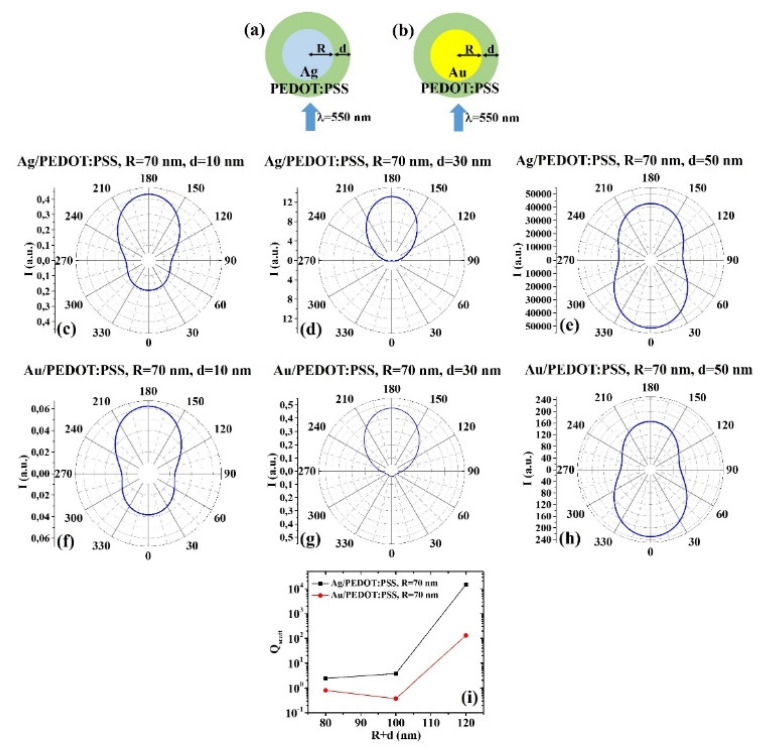
(**a**,**b**) Scheme of the structure of the simulated Ag/PEDOT:PSS (**a**) and Au/PEDOT:PSS (**b**) core–shell spherical particle with core radius R and shell width d and electromagnetic radiation of wavelength λ = 550 nm impinging on the particle from 0°. (**c**–**e**) Calculated polar diagrams for the intensity of the scattered light from the Ag/PEDOT:PSS core–shell spherical particle fixing the Ag core radius R to 70 nm and increasing the PEDOT:PSS shell width from 10 nm to 50 nm. (**f**–**h**) Calculated polar diagrams for the intensity of the scattered light from the Au/PEDOT:PSS core–shell spherical particle fixing the Au core radius R to 70 nm and increasing the PEDOT:PSS shell width from 10 nm to 50 nm. (**i**) Calculated scattering efficiency for the light (wavelength λ = 550 nm) scattering process of the Ag/PEDOT:PSS (black dots) and Au/PEDOT:PSS (red dots) core–shell spherical particles fixing the core radius to R = 70 nm and increasing the PEDOT:PSS shell width d from 10 nm to 50 nm.

The ScatLab software is, now, used to perform electromagnetic scattering simulations for the spherical bimetallic core–shell nanoparticles: in particular, an electromagnetic plan wave of wavelength λ = 550 nm is supposed to impinge from 0° on the single NP which is located in the origin of a reference system. Then, the ScatLab software is used to calculate the angular-dependent intensity I(θ) of the scattered electromagnetic wave and the scattering efficiency Q_scatt_. This is done for the spherical core–shell nanoparticles made by Ag/AZO, Au/AZO, Ag/ITO, Au/ITO, Ag/PEDOT:PSS, and Au/PEDOT:PSS for various combinations of the core radius R (30, 50, 70 nm) and shell width d (10, 30, 50 nm). The other input parameters are the values of n and k, as reported in Tab. 1. In each case, the results for the calculations of I(θ) are reported in polar diagrams and the results for the calculations of Q_scatt_ are reported in plots expressing the evolution of Q_scatt_ for each couple of metals when fixed R and increasing d. The results are reported in Figure 1, Figure 2, Figure 3, Figure 4, Figure 5, Figure 6, Figure 7, Figure 8, Figure 9 and Figure 10. In particular:

(1) Pure Ag and Au Particles

Figure 1 reports the scheme of the structure of the simulated Ag (Figure 1a) and Au (Figure 1b) pure spherical particle with radius R and electromagnetic radiation of wavelength λ = 550 nm impinging on the particle from 0°; in Figure 1c–e the calculated polar diagrams for the intensity of the scattered light from the Ag spherical particle changing the radius R from 30 nm to 70 nm; in Figure 1f–h the calculated polar diagrams for the intensity of the scattered light from the Au spherical particle changing the radius R from 30 nm to 70 nm; and in Figure 1i the calculated scattering efficiency for the light (wavelength λ = 550 nm) scattering process of the Ag (black dots) and Au (red dots) spherical particle increasing the particle radius from 30 nm to 70 nm.

For both Ag and Au particles, the scattering intensity at 180° increases, with respect to the scattering intensity at 0° (backscattered light), by increasing the radius of the particle (Figure 1c–h) and that the scattering efficiency is higher for the Ag particles than the Au particles in all the investigated size range (Figure 1i), whereas the scattering efficiency linearly increases with size. The increase of the scattering efficiency by increasing the size of a spherical particle is an obvious consequence of the Mie theory for which the dipole model can be used to approximate the scattering cross-sections of a particle with a diameter much smaller than the wavelength, λ, of the incident light: C_scatt_ = (1/6π)(2π/λ)^4^|α|^2^ with α the polarizability and for a spherical particle α = 3V(ε_p_−ε_m_/ε_p_+2ε_m_) and being V the volume of the particle, ε_p_ and ε_m_ the dielectric functions of the particle and of the surrounding environment, respectively. Hence, larger particles are predominantly scattering. 

(2) Ag/AZO and Au/AZO:

(a) R = 30 nm: Figure 2a,b reports the scheme of the structure of the simulated Ag/AZO (Figure 2a) and Au/AZO (Figure 2b) core–shell spherical particle with core radius R and shell width d and electromagnetic radiation of wavelength λ = 550 nm impinging on the particle from 0°; in Figure 2c–e the calculated polar diagrams for the intensity of the scattered light from the Ag/AZO core–shell spherical particle fixing the Ag core radius R to 30 nm and increasing the AZO shell width from 10 nm to 50 nm are reported; in Figure 2f–h the calculated polar diagrams for the intensity of the scattered light from the Au/AZO core–shell spherical particle fixing the Au core radius R to 30 nm and increasing the AZO shell width from 10 nm to 50 nm are presented; in Figure 2i the calculated scattering efficiency for the light (wavelength λ = 550 nm) scattering process of the Ag/AZO (black dots) and Au/AZO (red dots) core–shell spherical particles fixing the core radius to R = 30 nm and increasing the AZO shell width d from 10 nm to 50 nm is shown.

Fixing R = 30 nm and d = 10 nm (Figure 2c), we can see that the Ag/AZO system scatters light with higher intensity at 180° than at 0° and that the light scattering is, practically, confined between 210° and 150°. Correspondently, instead, the intensity of the light scattered by the Au/AZO system (Figure 2f) is zero at 0° and maximum at 180°; however, the scattered light is confined between 270° and 90°.

For R = 30 nm and d = 30 nm and R = 30 nm and d = 50 nm, the behavior of the Ag/AZO and Au/AZO systems is similar (Figure 2d,e,g,h), with the maximum of the intensity of the scattered light at 180°. However, in each condition, the Ag/AZO system if more efficient in scattering light at 180° than at 0°. Moreover, in any condition the scattering efficiency of the Ag/AZO system is higher than the scattering efficiency of the Au/AZO one (Figure 2i). However, the scattering efficiency of the Au/AZO system increases by increasing the particle size, while the scattering efficiency of the Ag/AZO system firstly decreases (d from 10 to 30 nm) then increases (d from 30 to 50 nm). 

(b) R = 50 nm: Figure 3a,b reports the scheme of the structure of the simulated Ag/AZO (Figure 3a) and Au/AZO (Figure 3b) core–shell spherical particle with core radius R and shell width d and electromagnetic radiation of wavelength λ = 550 nm impinging on the particle from 0°; in Figure 3c–e the calculated polar diagrams for the intensity of the scattered light from the Ag/AZO core–shell spherical particle fixing the Ag core radius R to 50 nm and increasing the AZO shell width from 10 nm to 50 nm are reported; in Figure 3f–h the calculated polar diagrams for the intensity of the scattered light from the Au/AZO core–shell spherical particle fixing the Au core radius R to 50 nm and increasing the AZO shell width from 10 nm to 50 nm are presented; in Figure 3i the calculated scattering efficiency for the light (wavelength λ = 550 nm) scattering process of the Ag/AZO (black dots) and Au/AZO (red dots) core–shell spherical particles fixing the core radius to R = 50 nm and increasing the AZO shell width d from 10 nm to 50 nm is shown. 

In this case, the polar diagrams show similar shape for the intensity of the scattered light from the Ag/AZO and Au/AZO systems apart for the condition R = 50 nm and d = 30 nm. In this last condition (Figure 3d,g), the intensity of light scattered by the Ag/AZO system (Figure 3d) is zero at 0° and maximum at 180°. In the same situation, the Au/AZO system (Figure 3g) scatters light with the best efficiency at 80°; however, the intensity is not zero at 0° and the minimum intensity for the scattered light is obtained between 60° and 90° and between 270° and 300°. Moreover, in any condition, the scattering efficiency of the Ag/AZO system is higher than the scattering efficiency of the Au/AZO one (Figure 3i). However, the scattering efficiency of the Ag/AZO system increases by increasing the particle size, while the scattering efficiency of the Au/AZO system firstly decreases (d from 10 to 30 nm) then increases (d from 30 to 50 nm).

(c) R = 70 nm: Figure 4a, b reports the scheme of the structure of the simulated Ag/AZO (Figure 4a) and Au/AZO (Figure 4b) core–shell spherical particle with core radius R and shell width d and electromagnetic radiation of wavelength λ = 550 nm impinging on the particle from 0°; in Figure 4c–e the calculated polar diagrams for the intensity of the scattered light from the Ag/AZO core–shell spherical particle fixing the Ag core radius R to 70 nm and increasing the AZO shell width from 10 nm to 50 nm are reported; in Figure 4f–h the calculated polar diagrams for the intensity of the scattered light from the Au/AZO core–shell spherical particle fixing the Au core radius R to 70 nm and increasing the AZO shell width from 10 nm to 50 nm are presented; in Figure 4i the calculated scattering efficiency for the light (wavelength λ = 550 nm) scattering process of the Ag/AZO (black dots) and Au/AZO (red dots) core–shell spherical particles fixing the core radius to R = 70 nm and increasing the AZO shell width d from 10 nm to 50 nm is shown.

In this case, the polar diagrams show similar shape for the intensity of the scattered light from the Ag/AZO and Au/AZO systems. However, a very different result is obtained in the condition R = 70 nm and d = 50 nm (Figure 4e,h) with respect to the other conditions (R = 70 nm and d = 10 nm, Figure 4c,f, and R = 70 nm and d = 30 nm, Figure 4d,g). In fact, for R = 70 nm and d = 10 nm (Figure 4c,f), R = 70 nm and d = 30 nm (Figure 4d,g), both the Ag/AZO and Au/AZO systems scatter light more efficiently at 180° than at 0°. This is particularly true in the case R = 70 nm and d = 30 nm (Figure 4d,g) for which the intensity of the scattered light is about zero at 0° and maximum at 180°. The opposite situation is, instead, established for R = 70 nm and d = 50 nm (Figure 4e,h): both systems scatter light with the maximum intensity at 0° (backscattering). The intensity of the scattered light is about zero at 180° for the Au/AZO system and, even if not exactly zero, very low for the Ag/AZO system. In addition, in this case, the Ag/AZO system has the higher overall scattering efficiency in all the investigated size range (Figure 4i). Moreover, the scattering efficiency of the Ag/AZO system increases by increasing the particle size, while the scattering efficiency of the Au/AZO system firstly decreases (d from 10 to 30 nm) then increases (d from 30 to 50 nm).2.3. Ag/ITO and Au/ITO

(3) Ag/ITO and Au/ITO: 

(a) R = 30 nm: Figure 5a, b reports the scheme of the structure of the simulated Ag/ITO (Figure 5a) and Au/ITO (Figure 5b) core–shell spherical particle with core radius R and shell width d and electromagnetic radiation of wavelength λ = 550 nm impinging on the particle from 0°; in Figure 5c–e the calculated polar diagrams for the intensity of the scattered light from the Ag/ITO core–shell spherical particle fixing the Ag core radius R to 30 nm and increasing the ITO shell width from 10 nm to 50 nm are reported; in Figure 5 f–h the calculated polar diagrams for the intensity of the scattered light from the Au/ITO core–shell spherical particle fixing the Au core radius R to 30 nm and increasing the ITO shell width from 10 nm to 50 nm are presented; in Figure 5i the calculated scattering efficiency for the light (wavelength λ = 550 nm) scattering process of the Ag/ITO (black dots) and Au/ITO (red dots) core–shell spherical particles fixing the core radius to R = 30 nm and increasing the ITO shell width d from 10 nm to 50 nm is shown.

In this case, the polar diagrams show similar shape for the intensity of the scattered light from the Ag/ITO and Au/ITO systems in all sizes conditions. It is interesting to note that the polar diagrams are practically identical for R = 30 nm and d = 10 nm (Figure 5c,f) and that, in this condition, the intensity of the scattered light is about zero at 0° and maximum at 180°. In the conditions R = 30 nm and d = 30 nm (Figure 5d,g), R = 30 nm and d = 50 nm (Figure 5e,h), both systems scatter light in any direction, however with maximum intensity at 180° and, in general, the Ag/ITO system with higher intensity at 180° with respect to 0°. In all these cases, however, the minimum intensity for the scattered light is obtained at 90° and 270°. For both systems the scattering efficiency (Figure 5i) increases by increasing the shell size and, in any condition, it is higher for the Ag/ITO system. 

(b) R = 50 nm: Figure 6a, b reports the scheme of the structure of the simulated Ag/ITO (Figure 6a) and Au/ITO (Figure 6b) core–shell spherical particle with core radius R and shell width d and electromagnetic radiation of wavelength λ = 550 nm impinging on the particle from 0°; in Figure 6c–e the calculated polar diagrams for the intensity of the scattered light from the Ag/ITO core–shell spherical particle fixing the Ag core radius R to 50 nm and increasing the ITO shell width from 10 nm to 50 nm are reported; in Figure 6f–h the calculated polar diagrams for the intensity of the scattered light from the Au/ITO core–shell spherical particle fixing the Au core radius R to 50 nm and increasing the ITO shell width from 10 nm to 50 nm are presented; in Figure 6i the calculated scattering efficiency for the light (wavelength λ = 550 nm) scattering process of the Ag/ITO (black dots) and Au/ITO (red dots) core–shell spherical particles fixing the core radius to R = 50 nm and increasing the ITO shell width d from 10 nm to 50 nm is shown. 

In addition, in this case, the polar diagrams show similar shape for the intensity of the scattered light from the Ag/ITO and Au/ITO systems in all sizes conditions, indicating a better efficiency of the Ag/ITO system in scattering light at 180° than at 0°. For example, for R = 50 nm and d = 30 nm (Figure 6d,g), the intensity of the light scattered at 0° by the Ag/ITO system is zero while it is not zero for the Au/ITO system. Concerning the overall scattering efficiency (Figure 6i), the scattering efficiency of the Ag/ITO system increases by increasing the particle size, while the scattering efficiency of the Au/ITO system firstly decreases (d from 10 to 30 nm) then increases (d from 30 to 50 nm). 

(c) R = 70 nm: Figure 7a, b reports the scheme of the structure of the simulated Ag/ITO (Figure 7a) and Au/ITO (Figure 7b) core–shell spherical particle with core radius R and shell width d and electromagnetic radiation of wavelength λ = 550 nm impinging on the particle from 0°; in Figure 7c–e the calculated polar diagrams for the intensity of the scattered light from the Ag/ITO core–shell spherical particle fixing the Ag core radius R to 70 nm and increasing the ITO shell width from 10 nm to 50 nm are reported; in Figure 7f–h the calculated polar diagrams for the intensity of the scattered light from the Au/ITO core–shell spherical particle fixing the Au core radius R to 70 nm and increasing the ITO shell width from 10 nm to 50 nm are presented; in Figure 7i the calculated scattering efficiency for the light (wavelength λ = 550 nm) scattering process of the Ag/ITO (black dots) and Au/ITO (red dots) core–shell spherical particles fixing the core radius to R = 70 nm and increasing the ITO shell width d from 10 nm to 50 nm is shown. 

In this case, for R = 70 nm and d = 10 nm (Figure 7c,f), the behavior of the Ag/ITO and Au/ITO particles is similar in scattering light, even if the Ag/ITO one more efficient in scattering light at 180° than at 0°. In addition, for R = 70 nm and d = 30 nm (Figure 7d,g) the behavior is similar; however, in this case, the intensity of the scattered light by the Ag/ITO particle at 0° is zero and maximum at 180° while the intensity of the scattered light by the Au/ITO particle is not zero at 0°, however low, and maximum at 180°. For R = 70 nm and d = 50 nm (Figure 7e,h), the intensity of the scattered light by the Au/ITO particle is about zero at 180° and maximum at 0°, for the Ag/ITO particle it is maximum at 0° and significant also at 180°, and minimum at 90° and 270°. Finally, the scattering efficiency of the Ag/ITO system increases (almost linearly) by increasing the particle size, while the scattering efficiency of the Au/ITO system firstly decreases (d from 10 to 30 nm) then increases (d from 30 to 50 nm). However, the Ag/ITO systems present a scattering efficiency higher in any size condition.

(4) Ag/PEDOT:PSS and Au/PEDOT:PSS:

(a) R = 30 nm: Figure 8a, b the scheme of the structure of the simulated Ag/PEDOT:PSS (Figure 8a) and Au/PEDOT:PSS (Figure 8b) core–shell spherical particle with core radius R and shell width d and electromagnetic radiation of wavelength λ = 550 nm impinging on the particle from 0°; in Figure 8c–e the calculated polar diagrams for the intensity of the scattered light from the Ag/PEDOT:PSS core–shell spherical particle fixing the Ag core radius R to 30 nm and increasing the PEDOT:PSS shell width from 10 nm to 50 nm are reported; in Figure 8f–h the calculated polar diagrams for the intensity of the scattered light from the Au/PEDOT:PSS core–shell spherical particle fixing the Au core radius R to 30 nm and increasing the PEDOT:PSS shell width from 10 nm to 50 nm are presented; in Figure 8i the calculated scattering efficiency for the light (wavelength λ = 550 nm) scattering process of the Ag/PEDOT:PSS (black dots) and Au/PEDOT:PSS (red dots) core–shell spherical particles fixing the core radius to R = 30 nm and increasing the PEDOT:PSS shell width d from 10 nm to 50 nm is shown.

In this case, it is interesting to note that for R = 30 nm and d = 10 nm (Figure 8c,f), both Ag/PEDOT:PSS and Au/PEDOT:PSS systems allow to obtain the maximum of the intensity for scattered light at 180° and the minimum at 0°; however, in this case, the Au/PEDOT:PSS system is more efficient I scattering light at 180° with respect to 0°. For R = 30 nm and d = 30 nm (Figure 8d,g), R = 30 nm and d = 50 nm (Figure 8e,h), the two systems scatter light similarly; however, the Ag/PEDOT:PSS system has better efficiency than the Au/PEDOT:PSS system at 180° than at 0°. The overall scattering efficiency (Figure 8i) increases, almost linearly, for both systems, in all the investigated size range and, in any condition, the scattering efficiency for the Ag/PEDOT:PSS systems is higher. 

(b) R = 50 nm: Figure 9a,b reports the scheme of the structure of the simulated Ag/PEDOT:PSS (Figure 9a) and Au/PEDOT:PSS (Figure 9b) core–shell spherical particle with core radius R and shell width d and electromagnetic radiation of wavelength λ = 550 nm impinging on the particle from 0°; in Figure 9c–e the calculated polar diagrams for the intensity of the scattered light from the Ag/PEDOT:PSS core–shell spherical particle fixing the Ag core radius R to 50 nm and increasing the PEDOT:PSS shell width from 10 nm to 50 nm are reported; in Figure 9f–h the calculated polar diagrams for the intensity of the scattered light from the Au/PEDOT:PSS core–shell spherical particle fixing the Au core radius R to 50 nm and increasing the PEDOT:PSS shell width from 10 nm to 50 nm are presented; in Figure 9i the calculated scattering efficiency for the light (wavelength λ = 550 nm) scattering process of the Ag/PEDOT:PSS (black dots) and Au/PEDOT:PSS (red dots) core–shell spherical particles fixing the core radius to R = 50 nm and increasing the PEDOT:PSS shell width d from 10 nm to 50 nm is shown.

In particular, for R = 50 nm and d = 10 nm (Figure 9c,f), R = 50 nm and d = 50 nm (Figure 9e,h), the polar diagrams show a similar behavior for the intensity of the light scattered by the Ag/PEDOT:PSS and Au/PEDOT:PSS particles, with the maximum intensity at 180°, the minimum at 90° and 270° and a low (even if not minimum) at 0°. More peculiar is the behavior for R = 50 nm and d = 30 nm (Figure 9d,g): in this case, the Ag/PEDOT:PSS system presents the maximum for the intensity of the scattered light at 180°, the minimum at 90° and 270° and a significant intensity at 0°; on the other hand, the Au/PEDOT:PSS system presents the maximum of the intensity of the scattered light at 0° (backscattered light) and the minimum at 180°. The overall scattering efficiency (Figure 9i) is about constant for the Ag/PEDOT:PSS particle for d from 10 nm to 30 nm then increases for d from 30 nm to 50 nm. Instead, it increases from d = 10 nm to d = 50 nm for the Au/PEDOT:PSS system. In any case, the scattering efficiency of the Ag/PEDOT:PSS particle is higher. 

(c) R = 70 nm: Figure 10a, b reports the scheme of the structure of the simulated Ag/PEDOT:PSS (Figure 10a) and Au/PEDOT:PSS (Figure 10b) core–shell spherical particle with core radius R and shell width d and electromagnetic radiation of wavelength λ = 550 nm impinging on the particle from 0°; in Figure 10c–e the calculated polar diagrams for the intensity of the scattered light from the Ag/PEDOT:PSS core–shell spherical particle fixing the Ag core radius R to 70 nm and increasing the PEDOT:PSS shell width from 10 nm to 50 nm are reported; in Figure 10f–h the calculated polar diagrams for the intensity of the scattered light from the Au/PEDOT:PSS core–shell spherical particle fixing the Au core radius R to 70 nm and increasing the PEDOT:PSS shell width from 10 nm to 50 nm are presented; in Figure 10i the calculated scattering efficiency for the light (wavelength λ = 550 nm) scattering process of the Ag/PEDOT:PSS (black dots) and Au/PEDOT:PSS (red dots) core–shell spherical particles fixing the core radius to R = 70 nm and increasing the PEDOT:PSS shell width d from 10 nm to 50 nm is shown. In this case, the polar diagrams shows similar results for the intensity of the light scattered by the Ag/PEDOT:PSS and Au/PEDOT:PSS particles in all the size conditions. In particular: for R = 70 nm and d = 10 nm (Figure 10c,f), both systems show the maximum intensity for the scattered light at 180°, the minimum at 90° and 270° and a significant intensity at 0°. However, the Ag/PEDOT:PSS system a more efficient in scattering light at 180° than at 0°. For R = 70 nm and d = 30 nm (Figure 10d,g), the intensity of the scattered light at 0° is almost zero and maximum at 180°. For R = 70 nm and d = 50 nm (Figure 10e,h), both systems show the maximum for the intensity of the scattered light at 0°, the minimum at 90° and 270° and a high intensity (even if not maximum) at 0°. Finally, the scattering efficiency of the Ag/PEDOT:PSS system increases by increasing the particle size, while the scattering efficiency of the Au/PEDOT:PSS system firstly decreases (d from 10 to 30 nm) then increases (d from 30 to 50 nm). However, the Ag/PEDOT:PSS systems present a scattering efficiency higher in any size condition.

Finally, the Mie’s theory, on which the present Scatlab calculations are based, is applicable only to spherical particles (spherical symmetry). Hence, the results of the present calculations are limited to such a situation; however, they could be a starting point to analyze the scattering properties of non-spherical particles. It can be observed that many natural and artificial small particles have nonspherical overall shapes or lack a spherically symmetric internal structure. It is now well recognized that the scattering properties of nonspherical particles can differ dramatically from those of “equivalent” Mie spheres. Therefore, the ability to accurately compute or measure light scattering by nonspherical particles in order to clearly understand the effects of particle nonsphericity on scattering patterns is very important [51]. In this regard, several techniques were developed for computing electromagnetic scattering by nonspherical particles based on numerically solving Maxwell’s equations [51,52]. The main classes of techniques can be, roughly, classified in: (a) the separation of variables method, (b) the discrete dipole approximation (a specific approach in a more general class called the volume-integral equation method), (c) the T-matrix approach, d) the Finite Difference Time Domain method. The basic idea of the separation of variables method is to make a separation ansatz for the solution to the scalar Helmholtz equation and to obtain a set of differential equations for each component function from the scalar Helmholtz equation. From the set of solution functions to these differential equations one can construct solenoidal vector wave functions that solve the vector Helmholtz equation [52]. The incident, scattered, and internal fields are expanded in suitable vector wave functions, and the expansion coefficients are determined by enforcing the boundary conditions on the particle surface. An important advantage of this method is its high numerical accuracy, making it a method suitable for benchmark computations [52]. A disadvantage of this approach is that for large size parameters of the spheroid and/or large refractive indices the system of linear equations to be solved becomes large, and ill-conditioning problems may occur. In the discrete dipole approximation, the effect of the exciting field is interpreted as inducing a dipole moment in each discrete volume cell [52]. This formalism leads to a system of linear equations that can be inverted numerically by standard techniques, such as Gaussian elimination or the conjugate gradient method. The computational complexity of the discrete dipole approximation depends on the method employed for solving the system of linear equations, and on suitable methods for reducing the large number of operations involved in calculating the matrix–vector products [52]. An important issue for this approach is how relate the relative permittivity to the polarizability in each cell. The T-matrix approach is based on the traditional description of the scattering problem, i.e., the computation of the scattered field for a given incident field. This procedure needs to be repeated for each new angle of incidence or each new form of the incident field. However, by contrast, the T-matrix approach offers a significantly more concise description of a particle’s scattering and absorption properties [52]. The objective in the development of the nullified method was to derive an approach suitable for numerically computing the scattered field in the exterior domain. The T-matrix is a quantity that contains (in approximate form) the full information about a particle’s optical properties at a given wavelength. It depends only on the particle’s size parameter, its shape, its refractive index, and on the particle’s orientation with respect to the coordinate system. The T matrix is independent of the incident field. The Finite Difference Time Domain method is the most direct method to solve Maxwell’s curl equations in differential form [52]. In this method both time and space are discretized, i.e., all spatial and temporal derivatives in Maxwell’s curl equations are replaced by finite difference quotients. Thus, the essence of this algorithm is to numerically solve an initial-value problem by marching a plane wave or pulse source, which is switched on at some initial time, through discrete time steps over a 5nite discretized spatial domain that includes the particle [52]. The main practical issue of this approach is the method of spatial discretization. 

## 3. Conclusions

In this work we reported calculations of angle-dependent light scattering intensity and scattering efficiency for Ag/AZO, Au/AZO, Ag/ITO, Au/ITO, Ag/PEDOT:PSS, and Au/PEDOT:PSS spherical core–shell nanoparticles by changing the core and shell sizes.

For various geometrical conditions the I(θ) diagram and scattering efficiency Q_scatt_ were calculated. Combining the I(θ) and Q_scatt_ informations, the best geometry for the bimetallic core–shell nanoparticles can be chosen for a specific application involving particular light scattering properties of the system. In general: the particles having the Ag core were found to have a higher scattering efficiency Q_scatt_ than the particles having the Au core. However, the strict comparison between the particles having the Ag core and the particles having the Au core was performed for any investigated R+d (core radius+shell thickness) condition by the evaluation of the corresponding I(θ) plots. In this way, for the two classes of systems, the intensity of the scattered light can be compared in any spatial direction so to choose the specific system having the best performance for a desired application based on direction-dependent light scattering, as in plasmonic solar cells exploiting the light scattering properties of Ag and Au nanoparticles in contact to or embedded in AZO, ITO, PEDOT:PSS layers.

## Figures and Tables

**Table 1:** Values for the real part, n, and imaginary part, k, of the refractive index of the materials composing the core–shell particles (corresponding to the electromagnetic radiation wavelength λ = 550 nm) and used for the calculations [50].

λ = 550 nm	Au	Ag	AZO	ITO	PEDOT:PSS
n	0.42415	0.059582	1.8559	1.9251	1.5155
k	2.4721	3.5974	0.0016581	0.0021684	0.0075967

## Data Availability

All the research data are presented within this article.

## References

[1] Murray W.A., Barnes W.L. (2007). Plasmonic Materials. Adv. Mater..

[2] Odom T., Schatz G.C. (2011). Introduction to Plasmonics. Chem. Rev..

[3] Mayer S.A. (2007). Plasmonics: Fundamentals and Applications.

[4] Xiong Y., Lu X. (2015). Metallic Nanostructures: From Controlled Synthesis to Applications.

[5] Quinten M. (2011). Optical Properties of Nanoparticle Systems.

[6] Ruffino F., Crupi I., Simone F., Grimaldi M.G. (2011). Formation and evolution of self-organized Au nanorings on indium-tin-oxide surface. Appl. Phys. Lett..

[7] Ruffino F., Pugliara A., Carria E., Romano L., Bongiorno C., Fisicaro G., La Magna A., Spinella C., Grimaldi M. (2012). Towards a laser fluence dependent nanostructuring of thin Au films on Si by nanosecond laser irradiation. Appl. Surf. Sci..

[8] Ruffino F., Grimaldi M.G. (2019). Nanostructuration of Thin Metal Films by Pulsed Laser Irradiations: A Review. Nanomaterials.

[9] Gentile A., Ruffino F., Grimaldi M.G. (2016). Complex-Morphology Metal-Based Nanostructures: Fabrication, Characterization, and Applications. Nanomaterials.

[10] Mohapatra S., Mishra Y., Ghatak J., Kabiraj D., Avasthi D.K. (2008). Surface plasmon resonance of Ag nanoparticles embedded in partially oxidized amorphous Si matrix. J. Nanosci. Nanotechnol..

[11] Kumar M., Sandeep C.S.S., Kumar G., Mishra Y., Philip R., Reddy G.B. (2013). Plasmonic and Nonlinear Optical Absorption Properties of Ag:ZrO_2_ Nanocomposite Thin Films. Plasmonics.

[12] Mishra Y.K., Mohapatra S., Singhal R., Avasthi D.K., Agarwal D.C., Ogale S.B. (2008). Au–ZnO: A tunable localized surface plasmonic nanocomposite. Appl. Phys. Lett..

[13] Atwater H.A., Polman A. (2010). Plasmonics for improved photovoltaic devices. Nat. Mater..

[14] Mandal P., Sharma S. (2016). Progress in plasmonic solar cell efficiency improvement: A status review. Renew. Sustain. Energy Rev..

[15] Araújo A., Mendes M.J., Mateus T., Costa J., Nunes D., Fortunato E., Águas H., Martins R. (2018). Ultra-fast plasmonic back reflectors production for light trapping in thin Si solar cells. Sol. Energy.

[16] Nasser H., Saleh Z.M., Özkol E., Günoven M., Bek A., Turan R. (2013). Fabrication of Ag Nanoparticles Embedded in Al:ZnO as Potential Light-Trapping Plasmonic Interface for Thin Film Solar Cells. Plasmonics.

[17] Huang M., Hameiri Z., Gong H., Wong W.-C., Aberle A.G., Mueller T. (2014). Hybrid silver nanoparticle and transparent conductive oxide structure for silicon solar cell applications. Phys. Status Solidi RRL Rapid Res. Lett..

[18] Santbergen R., Temple T.L., Liang R., Smets A.H.M., Swaaij R.A.C.M.M.V., Zeman M. (2012). Application of plasmonic silver island films in thin-film silicon solar cells. J. Opt..

[19] Shirshneva-Vaschenko E.V., Sokura A.L., Baidakova M.V., Yagovkina A.M., Snezhnaia Z.G., Shirshnev P.S., Romanov E.A. (2019). Study of the influence of the ZnO:Al polycrystalline film morphology on the silver nanoparticles formation. J. Phys. Conf. Ser..

[20] Malek G.A., Aytug T., Liu Q., Wu J.Z. (2015). Plasmonic Three-Dimensional Transparent Conductor Based on Al-Doped Zinc Oxide-Coated Nanostructured Glass Using Atomic Layer Deposition. ACS Appl. Mater. Interfaces.

[21] Mendes M.J., Morawiec S., Mateus T., Lyubchyk A., Águas H., Ferreira I., Fortunato E., Martins R., Priolo F., Crupi I. (2015). Broadband light trapping in thin film solar cells with self-organized plasmonic nano-colloids. Nanotechnology.

[22] Chuang S.-H., Tsung C.-S., Cheng-Sheng T., Ou S.-L., Horng R.-H., Lin C.-Y., Wuu D.-S. (2015). Transparent Conductive Oxide Films Embedded with Plasmonic Nanostructure for Light-Emitting Diode Applications. ACS Appl. Mater. Interfaces.

[23] Mirzaee M., Dolati A. (2014). Effect of content silver and heat treatment temperature on morphological, optical, and electrical properties of ITO films by sol–gel technique. J. Nanoparticle Res..

[24] Islam K., Chowdhury F.I., Okyay A.K., Nayfeh A. (2015). Comparative study of thin film n-i-p a-Si:H solar cells to investigate the effect of absorber layer thickness on the plasmonic enhancement using gold nanoparticles. Sol. Energy.

[25] Grochowska K., Siuzdak K., Karczewski J., Śliwiński G. (2015). Functionalization of indium-tin-oxide electrodes by laser-nanostructured gold thin films for biosensing applications. Appl. Surf. Sci..

[26] Alkhalayfeh M.A., Aziz A.A., Pakhuruddin M.Z. (2021). An overview of enhanced polymer solar cells with embedded plasmonic nanoparticles. Renew. Sustain. Energy Rev..

[27] Kalfagiannis N., Karagiannidis P., Pitsalidis C., Hastas N., Panagiotopoulos N., Patsalas P., Logothetidis S. (2014). Performance of hybrid buffer Poly(3,4-ethylenedioxythiophene) poly(styrenesulfonate) layers doped with plasmonic silver nanoparticles. Thin Solid Films.

[28] Sutradhar P., Saha M. (2016). Silver Nanoparticles: Synthesis and Its Nanocomposites for Heterojunction Polymer Solar Cells. J. Phys. Chem. C.

[29] Xie F.-X., Choy W.C.H., Wang C.C.D., Sha W.E.I., Fung D.D.S. (2011). Improving the efficiency of polymer solar cells by incorporating gold nanoparticles into all polymer layers. Appl. Phys. Lett..

[30] Notarianni M., Vernon K., Chou A., Aljada M., Liu J., Motta N. (2014). Plasmonic effect of gold nanoparticles in organic solar cells. Sol. Energy.

[31] Said D., Ali A., Khayyat M., Boustimi M., Loulou M., Seoudi R. (2019). A study of the influence of plasmonic resonance of gold nanoparticle doped PEDOT: PSS on the performance of organic solar cells based on CuPc/C60. Heliyon.

[32] Susanti E., Wulandari P. (2018). Herman Effect of localized surface plasmon resonance from incorporated gold nanoparticles in PEDOT:PSS hole transport layer for hybrid solar cell applications. J. Physics: Conf. Ser..

[33] Truong N.T.N., Kim C.D., Reddy V.R.M., Thai V.H., Jeon H.J., Park C. (2020). Shape control of plasmonic gold nanoparticles and its application to vacuum-free bulk hetero-junction solar cells. J. Mater. Sci. Mater. Electron..

[34] Franken R.H., Stolk R.L., Li H., Van Der Werf C.H.M., Rath J.K., Schropp R.E. (2007). Understanding light trapping by light scattering textured back electrodes in thin film n-i-p-type silicon solar cells. J. Appl. Phys..

[35] Ginley D.S., Hosono H., Paine D.C. (2010). Handbook of Transparent Conductors.

[36] Ruffino F., Pugliara A., Carria E., Bongiorno C., Grimaldi M. (2013). Light scattering calculations from Au and Au/SiO2 core/shell nanoparticles. Phys. E Low-Dimens. Syst. Nanostructures.

[37] Ruffino F. (2021). Light-Scattering Simulations from Spherical Bimetallic Core–Shell Nanoparticles. Micromachines.

[38] Mie G. (1908). Beiträge zur Optik trüber Medien, speziell kolloidaler Metallösungen. Ann. der Phys..

[39] Van De Hulst H.C., Twersky V. (1957). Light Scattering by Small Particles.

[40] Wriedt T. (2009). Light scattering theories and computer codes. J. Quant. Spectrosc. Radiat. Transf..

[41] Mishchenko M.I., Travis L.D., Lacis A.A. (2002). Scattering, Absorption, and Emission of Light by Small Particles.

[42] Huxley A.F. (1968). A Theoretical Treatment of the Reflexion of Light by Multilayer Structures. J. Exp. Biol..

[43] Small A., Hong S., Pine D. (2005). Scattering properties of core-shell particles in plastic matrices. J. Polym. Sci. Part B Polym. Phys..

[44] https://scattport.org/index.php/light-scattering-software?start=100.

[45] Bazhan V. SCATLAB Version 1.2.0.111. http://www.scatlab.com/index.html.

[46] Sowa Y., Steel B.C., Berry R.M. (2010). A simple backscattering microscope for fast tracking of biological molecules. Rev. Sci. Instrum..

[47] Siems A., Weber S., Boneberg J., Plech A. (2011). Thermodynamics of nanosecond nanobubble formation at laser-excited metal nanoparticles. New J. Phys..

[48] Tuoriniemi J., Johnsson A.-C.J.H., Perez Holmberg J., Gustafsson S., Gallego-Urrea J.A., Olsson E., Pettersson J.B.C., Hassellöv M. (2014). Intermethod comparison of the particle size distributions of colloidal silica nanoparticles. Sci. Technol. Adv. Mater..

[49] Dmitruk I.M., Malynych S.Z., Grabovskyi E.S., Kravets V.K., Pinchuk A.O. (2014). Light scattering by silver nanoparticles in col-loidal solutions for improved photovoltaics devices. Proc. NAP.

[50] https://www.refractiveindex.info.

[51] Mishchenko M.I., Hovenier J.W., Travis L.D. (2000). Light Scattering by Nonspherical Particles-Theory, Measurements, and Applications.

[52] Kahnert F.M. (2003). Numerical methods in electromagnetic scattering theory. J. Quant. Spectrosc. Radiat. Transf..

